# Endovascular Repair of Blunt Aortic Trauma: A Multidisciplinary Approach and a Retrospective Multicenter Study

**DOI:** 10.3390/jcm15010068

**Published:** 2025-12-22

**Authors:** Ilenia Di Sario, Enrico Franceschini, Emanuele Gatta, Gabriele Pagliariccio

**Affiliations:** 1 Vascular Surgery Unit, Ospedale Giuseppe Mazzini, 64100 Teramo, Italy; 2 Vascular Surgery Unit, Ospedali Riuniti Torrette, 60126 Ancona, Italy

**Keywords:** blunt traumatic thoracic aortic injury, TEVAR, cerebrospinal fluid drainage

## Abstract

**Background/Objectives:** Blunt traumatic thoracic aortic injury is a rare but often fatal condition, typically resulting from high-energy deceleration mechanisms such as motor vehicle collisions or falls from height. Mortality can reach 80–90% at the scene, with in-hospital mortality up to 46%. Early diagnosis and appropriate management are essential to improve outcomes. **Methods:** We retrospectively analyzed 45 patients (29 males, 16 females) with varying degrees of aortic isthmus injury treated between January 2007 and December 2024 at two Italian vascular surgery centers. Aortic lesions were graded 0–3, with 40 patients undergoing thoracic endovascular aortic repair. Procedures utilized Gore TAG or Medtronic Valiant endografts. Perioperative management included controlled hypotension and left subclavian artery coverage was performed when necessary. **Results**: Procedural success was achieved in all patients (100%), with one type II endoleak. No perioperative complications or spinal cord ischemia were observed. Coverage of the left subclavian artery was required in 28.9% of cases. Thirty-day mortality was 9%, with no deaths directly attributable to aortic injury. Postoperative CTA confirmed adequate endograft placement in all patients. **Conclusions:** Endovascular repair is a safe and effective approach for BTAI, with excellent short-term outcomes. Management should be tailored to injury severity and patient comorbidities, with ongoing vigilance for spinal cord ischemia.

## 1. Introduction

Blunt traumatic thoracic aortic injury (BTAI) represents the second leading cause of death from blunt trauma after head injury [1]. It is associated with 80–90% mortality at the accident scene [2]. Of those reaching the hospital, mortality can still reach 32% within the first 24 h, with about one-third of cases dying before surgical repair can be performed [3,4,5]. The overall incidence of BTAI ranges between 1.5% and 2% among patients admitted for chest trauma [6].

Most injuries result from car accidents (70%), followed by motorcycle crashes (13%) and falls from height (7%). However, only about 1% of patients involved in motor vehicle collisions present with BTAI. These data may be underestimated, as they do not include patients who die at the scene [7].

BTAI mainly occurs at the level of the aortic isthmus, although the underlying reason still remains incompletely understood.

Regardless of the proposed mechanisms, aortic injury develops in two stages: rupture of the intima and media, followed by disruption of the adventitia. The damage may be partial or circumferential.

BTAI can be classified into four types [1]:

1. Type I: intimal tear
2. Type II: intramural hematoma
3. Type III: pseudoaneurysm
4. Type IV: rupture

The clinical symptoms are nonspecific and depend on lesion severity. Patients with low-grade injuries may be asymptomatic or may present with retro-scapular pain; more severe cases can manifest with hemorrhagic shock.

Clinical signs may include chest wall injury, pseudo-coarctation syndrome, a systolic murmur, or paraplegia. The severity of symptoms primarily reflects the traumatic mechanism. Indicators of significant vascular injury—“hard signs”—include active hemorrhage, pulsatile hematoma, bruit or thrill, absence of distal pulses, and limb ischemia.

However, there are no specific clinical signs for BTAI, making thorough physical examination essential, particularly in the presence of chest wall deformities (fractures, crepitus), bruising, penetrating injuries, tracheal deviation, or reduced breath sounds suggesting hemothorax or pneumothorax.

After the general assessment, a detailed vascular evaluation is crucial, particularly assessing central and peripheral pulses.

Associated abdominal or thoracic injuries, hypotension, and lack of restraint during motor vehicle crashes have been identified as predictors of BTAI.

In most polytrauma patients, the diagnosis is primarily radiological because total-body computed tomography angiography (CTA) is routinely performed to detect concomitant injuries. CTA is fast, reproducible, and has near-100% sensitivity and specificity for identifying BTAI.

CTA findings associated with BTAI include mediastinal hematoma, hemothorax, pseudoaneurysm, altered aortic contour, and intimal flap or thrombus.

All patients with suspected BTAI should be transferred to a higher-level trauma center.

The optimal timing of intervention in BTAI remains debated. Many ruptures occur within the first 24 h, leading to historically favored immediate repair. Recent studies, however, indicate lower paralysis and mortality rates with delayed repair.

Nonoperative management (NOM) can be used as a bridge strategy to stabilize polytrauma patients and relies on strict clinical and radiological monitoring [8,9,10,11,12]. A 2008 prospective multicenter AAST study showed similar complication rates but significantly higher mortality in early repair; delayed repair was associated with better outcomes in patients with multiple injuries [12].

NOM is generally recommended for grade I and II injuries under strict surveillance.

For grade III injuries (pseudoaneurysm), the need for NOM or immediate repair depends on several factors. Early intervention is recommended when two or more of the following are present: (1) shock (lactate > 4 mmol/L), (2) posterior mediastinal hematoma > 10 mm, (3) pseudoaneurysm-to-aorta diameter ratio > 1.4.

Lesion type remains the main determinant for treatment timing. Patients with an arch hematoma ≥ 15 mm are at significantly higher risk of death from BTAI compared to those with smaller hematomas. In contrast, minimal aortic injuries (intimal tears), occurring in about 10% of cases, may be managed conservatively with serial imaging [13].

Optimal perioperative management by an experienced trauma team is essential to determine treatment priorities. Excessive fluid administration should be avoided, as it may worsen bleeding, coagulopathy, or rupture. In patients with severe brain or lung injuries, delayed repair is preferred to reduce neurologic or respiratory deterioration [14].

BTAI treatment options include open repair (OR) or endovascular repair (TEVAR), the latter being the most widely used today [10,11,12,15,16].

Compared with OR, TEVAR shows lower mortality (9.7% vs. 27.7%; *p* < 0.001) and a trend toward reduced paralysis (0.4% vs. 2.9%) [16].

## 2. Materials and Methods

Between January 2007 and December 2024, a total of 45 patients (29 male and 16 female), with a mean age of 50 ± 3 and varying degrees of isthmic aortic rupture, were treated at the Vascular Surgery Units of Mazzini Hospital in Teramo and at the Azienda Ospedaliero-Universitaria of Ancona. More specifically, 5 patients presented with grade 1 aortic involvement, 12 with grade 2, 25 with grade 3, and 3 with grade 4 injuries involving complete aortic rupture.

At admission to the emergency department, 11 patients (24.4%) were in hemorrhagic shock.

Isolated aortic trauma was identified in 26.7% of cases (12 patients), while the remaining 74.3% (33 patients) had concomitant injuries affecting other vascular or anatomical districts. 30 patients (66.7%) had concomitant orthopedic injuries, 17 patients (37.8%) had abdominal injuries, 3 (6.7%) had thoracic injuries, and 8 (17.8%) had neurosurgical injuries. Some patients presented with multiple types of injuries simultaneously.

Of these 45 patients, 40 underwent endovascular treatment, and five patients were managed conservatively with blood pressure control and serial follow-up CTA scans.

All procedures were performed under general anesthesia in all patients. Informed consent was obtained from those who were not in hemorrhagic shock, whereas in the remaining cases the intervention was conducted under emergency conditions without prior consent (Table 1).

Data were processed using Microsoft Excel 365 (version 2402, Microsoft Corporation, Redmond, WA, USA).

## 3. Results

All procedures were performed under general anesthesia. In every case, vascular access for endograft deployment and release was obtained surgically.

In 28 (62.2%) patients the Medtronic Captivia endograft was used, whereas in 17 (37.8%) patients the Gore TAG endograft was employed, with an oversizing ranging between 20% and 30%.

Endograft deployment was performed using a controlled hypotension technique to ensure maximum precision. In 13 patients (28.9%), coverage of the left subclavian artery was required to extend the proximal sealing zone.

The procedural success rate was 100%, with one case a single case of type II endoleak with no type I endoleak. None of the patients experienced perioperative complications, and no cases of spinal cord ischemia were observed. One patient experienced a perioperative stroke. In-hospital mortality was 13.3% (6 patients) and was not related to the aortic pathology.

**Figure 1:**
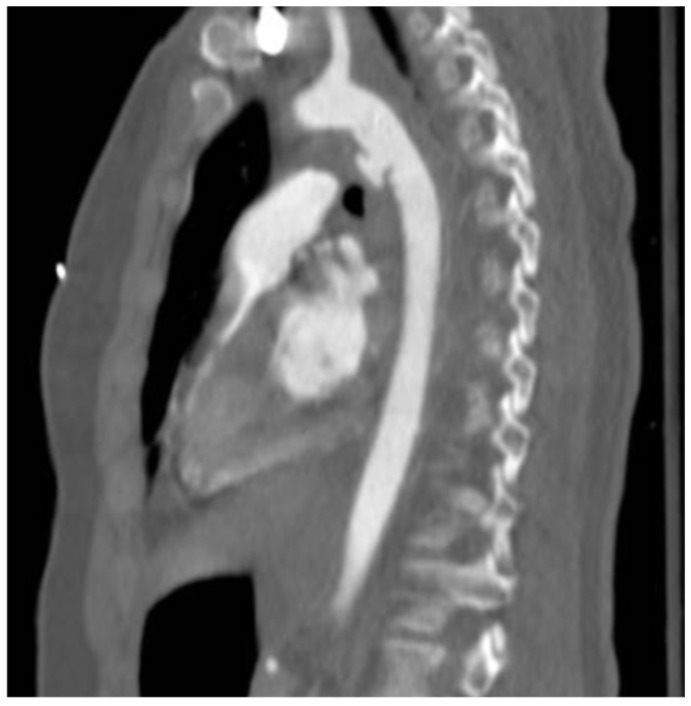
CTA showed type 3 BTAI.

**Figure 2:**
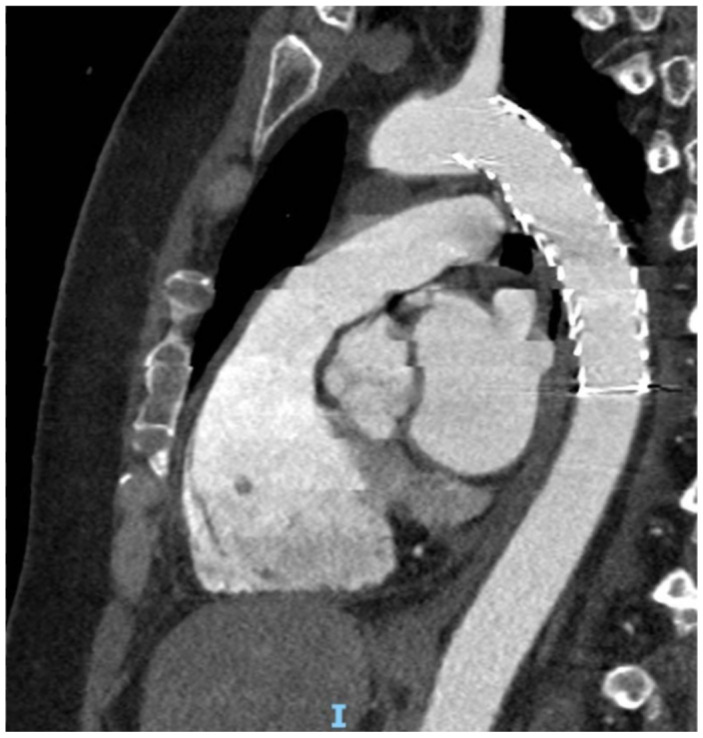
Post-operative CTA showed correct positioning and patency of the endoprosthesis (I: With complete exclusion of aortic rupture and complite obliteration of the tract).

All patients underwent a CT angiography (CTA) 24 h after the procedure (Figure 1 and Figure 2).

At 30 days, the mortality rate was 8.9% (4 patients); however, in none of these cases was the death attributable to the aortic injury (Table 2).

## 4. Discussion

Blunt thoracic aortic injuries (BTAI) represent an extremely rare but often fatal condition, with a mortality rate of approximately 32% within the first 24 h. When treatment is delayed, mortality may reach as high as 90% [17]. Therefore, prompt diagnosis is essential to improve outcomes in patients with suspected aortic trauma.

BTAIs are generally caused by acceleration–deceleration mechanisms and therefore typically occur in motor vehicle collisions or falls from height. The rupture most commonly occurs at the aortic isthmus, due to the specific unique mechanical forces acting at this level [1].

Historically, these injuries were treated with open surgery, which was associated with a high rate of complications, particularly in polytrauma patients. The advent of endovascular techniques has led to a profound change in management, making TEVAR the treatment of choice in most cases of BTAI [18,19,20].

Open repair via left thoracotomy has historically been associated with high mortality (16–31%), stroke (4–8%), and paraplegia (5–19%) [10,11,15].

Endovascular repair is now considered the preferred approach. It requires adequate proximal and distal landing zones (≥20 mm each), with diameters between 16 and 42 mm. The endograft should be oversized by 10–20%. Hypovolemia may reduce vessel diameter by up to 40%, increasing the risk of undersizing (type I endoleak, 4.2%) [21] or oversizing (device infolding or collapse, 1.2%) [16]. To reduce sizing errors, the aortic root may be used as a more reliable reference diameter in young trauma patients.

When the distance between the lesion and the left subclavian artery (LSA) origin is <15 mm, a proper seal is not feasible, and intentional LSA coverage is required in ~30% of cases [12,13,14,15,16,21]. LSA revascularization is recommended in patients with left internal mammary–to-coronary bypass, dominant left vertebral artery, hypoplastic right vertebral artery, or incomplete circle of Willis20. Newer techniques (chimney/periscope/sandwich, in situ fenestration, branched devices) now allow extension of the proximal landing zone.

In BTAI, TEVAR with 10–15 cm devices is typically performed. Routine cerebrospinal fluid (CSF) drainage is not indicated unless the distal thoracic aorta is extensively covered or the patient has prior aortic interventions. CSF drainage is usually performed only in cases of postoperative neurological deficits.

Several aspects of management, however, remain debated. The optimal timing of intervention and the severity thresholds requiring treatment are still under discussion. In general, urgent treatment is required for the most severe injuries, whereas for grade III lesions, intervention may be delayed for up to 24 h while monitoring the injury with CTA and stabilizing the patient, who often has additional traumatic injuries [12,13,14].

A multidisciplinary approach is of fundamental importance, with the anesthesiologist–intensivist playing a central role. Once the radiological diagnosis is confirmed, the patient is managed by the intensive care team, who ensure placement of large-bore venous access for the administration of fluids and medications [9]. The primary goal is to maintain SBP < 100 mmHg and HR < 100 bpm to prevent worsening of the injury. Esmolol is the drug of choice; if contraindicated or ineffective, calcium channel blockers, nitroglycerin, or sodium nitroprusside may be used. Observational studies have demonstrated that antihypertensive therapy and negative inotropes are associated with improved early mortality and morbidity [9]. Without strict blood pressure control, the risk of rupture in patients with contained injuries is approximately 12%, decreasing to 1.5–2% with adequate management [22]. The main resuscitation challenge is balancing hypertension, which may worsen the aortic injury, and hypotension, which may aggravate neurological injury.

Another key aspect is whether cerebrospinal fluid (CSF) drainage should be performed to prevent spinal cord ischemia (SCI). Current evidence suggests that routine CSF drainage is no longer recommended when the endograft length is <15 cm, as the risk of SCI is like the risk of CSF catheter–related complications. CSF drainage remains reserved for selected cases, such as patients with prior aortic surgery or when the distal third of the descending thoracic aorta is covered. It is typically performed postoperatively in patients with new neurological deficits [23,24,25].

Despite the relatively small sample size of our series, our findings provide meaningful insights. Certain strategies can minimize perioperative risk. Total-body CTA is essential to identify associated injuries—especially cerebral trauma—to determine priorities and treatment timing. Controlled hypotension, both pre- and intraoperatively, remains crucial [1].

However, once correct endograft positioning and exclusion of type I endoleaks are confirmed, it becomes essential to transition from controlled hypotension to controlled hypertension to ensure adequate spinal cord perfusion and reduce the risk of SCI.

In agreement with the literature, our experience involved two devices: the Gore TAG, featuring a repositionable delivery system allowing correction of device orientation even in sharply curved arches, and the Medtronic Captivia, which incorporates proximal anchoring hooks suitable for shorter landing zones.

The choice of endograft depends on several factors, including lesion site and length, aortic diameter, the position of the left subclavian artery relative to the injury, and whether coverage is required. Device selection is essential not only for immediate technical success but also for postoperative and long-term outcomes [16,20,26].

The main limitation of this study is the relatively small cohort. Nevertheless, our management aligns with current evidence. As highlighted by Estrera [10], a multidisciplinary approach with continuous ICU involvement is essential for optimal hemodynamic control, especially in polytrauma patients. Similarly, Hemmila [11] emphasized individualized timing of intervention, determined by lesion stability, associated injuries, and overall clinical status. Consistent with the broader literature, endovascular repair remains the preferred treatment option [16,17,26], as it is less invasive, allows earlier intervention, reduces blood loss compared with open repair, and ensures excellent perioperative and long-term results.

## 5. Conclusions

Although our experience is limited, we draw conclusions based on current guidelines, and our findings remain consistent with current guideline recommendations. Endovascular repair is currently the treatment of choice for BTAI, offering improved perioperative and postoperative outcomes compared with open repair.

The primary concern remains spinal cord ischemia (SCI), which continues to represent an unresolved issue with no definitive consensus on prevention or management strategies.

The timing and indication for treatment in patients with type II and type III BTAI should therefore be individualized, considering lesion characteristics, associated injuries, and the overall clinical condition of the patient.

## Figures and Tables

**Table 1:** Demographic and BTAI characteristics.

	BTAI (n. 45)
Age	50 ± 3
Male	29 (64.4%)
Type I lesion	5 (11.1%)
Type II lesion	12 (26.7%)
Type III lesion	25 (55.5%)
Type IV lesion	3 (6.7%)
Hemorrhagic shock	11 (24.4%)
Isolated lesion	12 (26%)
Orthopedics injuries	30 (66.7%)
Abdominal injuries	17 (37.8%)
Thoracic injuries	3 (6,7%)
Neurosurgical injuries	8 (17.8%)
Endovascular treatment	40 (88.8%)

**Table 2:** Intraprocedural and perioperative data.

	BTAI (n. 45)
General Anesthesia	45 (100%)
Medtronic Captivia	28 (62.2%)
Gore TAG	17 (37%)
LSA Coverage	13 (28.9%)
Procedural success rate	45 (100%)
Type I Endoleak	0
Type II Endoleak	1 (2.2%)
SCI	0
STROKE	1 (2.2%)
In Hospital Mortality (not aortic related)	6 (13.3%)

## Data Availability

The data presented in this study are available on request from the corresponding author. The data are not publicly available due to privacy restrictions.

## Abbreviations

The following abbreviations are used in this manuscript:

BTAI	Blunt traumatic thoracic aortic injury
CTA	Computed Tomography angiography
NOM	Nonoperative management
AAST	American Association for the Surgery of Trauma
OR	open repair
TEVAR	endovascular repair
CSF	cerebrospinal fluid
SCI	spinal cord ischemia
